# Correspondence

**Published:** 1870-10

**Authors:** Theodore Griffin

**Affiliations:** 582 South State Street


### Correspondence.

PROF. DAVIS:—Is it true that the practice of homœopathy is interdicted in Russia?

Some months ago, I read a statement in some of our medical journals, that the Emperor of Russia, after giving homœopathy a fair and impartial trial, for three years, in hospitals set apart for that purpose, in St. Petersburg, had issued a decree that homœopathy should not be practiced in any of the dominions of Russia, under penalty of fine and imprisonment. Will you be kind enough to give us the truth of this matter, in as full a manner as possible, and oblige many who wish to use this fact as a climax, and as a *clincher* to our anti-homœopathic arguments, with the deluded admirers of the *similia similibus curantur* vaguery, the champion system of small ideas—unexampled in their narrowness—and of small doses, microscopic in their dimensions.

Yours, truly,

THEODORE GRIFFIN, M.D.,

*582 South State Street.*

